# Crystal structure of the tri­ethyl­ammonium salt of 3-[(4-hy­droxy-3-meth­oxy­phen­yl)(4-hy­droxy-2-oxo-2*H*-chromen-3-yl)meth­yl]-2-oxo-2*H*-chromen-4-olate

**DOI:** 10.1107/S2056989018001561

**Published:** 2018-02-02

**Authors:** Muhammad Ikram, Sadia Rehman, Afzal Khan, Carola Schulzke

**Affiliations:** a Department of Chemistry, Abdul Wali Khan University Mardan, Pakistan; b Department of Microbiology, Abbotabad University of Science and Technology, Abbotabad, Pakistan; c Institut für Biochemie, Ernst-Moritz-Arndt Universität Greifswald, Felix-Hausdorff-Strasse 4, D-17487 Greifswald, Germany

**Keywords:** crystal structure, 4-hy­droxy-3-meth­oxy­phenyl dicoumarol, 4-hy­droxy­coumarin derivatives, negative charge-assisted hydrogen bonds, short intra­molecular hydrogen bonds

## Abstract

The reaction between 3,3′-[(3-meth­oxy-4-hy­droxy­phen­yl)methanedi­yl]bis­(4-hy­droxy-2*H*-chromen-2-one) and tri­ethyl­amine in methanol yielded the title compound tri­ethyl­ammonium 3-[(4-hy­droxy-3-meth­oxy­phen­yl)(4-hy­droxy-2-oxo-2*H*-chromen-3-yl)meth­yl]-2-oxo-2*H*-chromen-4-olate, C_6_H_16_N^+^·C_26_H_17_O_8_^−^ or (NHEt_3_)^+^(C_26_H_17_O_8_)^−^, which crystallized directly from its methano­lic mother liquor. The non-deprotonated coumarol substituent shares its H atom with the deprotonated coumarolate substituent in a short negative charge-assisted hydrogen bond in which the freely refined H atom is moved from its parent O atom towards the acceptor O atom, elongating the covalent O—H bond to 1.18 (3) Å. The respective H atom can therefore be described as being shared by two alcohol O atoms, culminating in the formation of an eight-membered ring.

## Abstract 2

3,3′-[(3-Meth­oxy-4-hy­droxy­phen­yl)methanedi­yl]bis­(4-hy­droxy-2*H*-chromen-2-one), the 4-hy­droxy-3-meth­oxy­phenyl-substituted derivative of dicoumarol, was deprotonated by the addition of tri­ethyl­amine, yielding the respective ammonium salt which was crystallized from a methanol solution. The deprotonated dicoumarol derivative exhibits an intra­molecular negative charge-assisted hydrogen bond between the deprotonated and non-deprotonated alcohol functions of the coumarol substituents.

## Chemical context   

Requisite chemotherapeutical treatments of cancer and inhibition of bacterial activities encourage the design of drugs that can effectively target the affected cells or respective pathogens (Nolan *et al.*, 2007 ▸; Jung & Park, 2009 ▸).

4-Hy­droxy coumarine and its derivatives have been developed and exploited by various researchers in this context (Nolan *et al.*, 2007 ▸; Tavolari *et al.*, 2008 ▸; Jung & Park, 2009 ▸; Li *et al.*, 2015 ▸; David, 2017 ▸). In biological tests with 3,3′-[(3-meth­oxy-4-hy­droxy­phen­yl)methanedi­yl]bis­(4-hy­droxy-2*H*-chromen-2-one), much lower than expected cytotoxic activity was found (Rehman *et al.*, 2013 ▸), which may be attributed to insufficient solubility. The hydro­phobic nature of this compound is most likely due to strong intra­molecular hydrogen bonding between the two coumarol moieties *via* two O—H⋯O=C inter­actions, which was confirmed for the solid state by X-ray structural analysis of this compound (Bandyopadhyay, 2015 ▸) and close relatives (Manolov *et al.*, 2006 ▸; Stanchev *et al.*, 2007 ▸).

Hydro­phobic mol­ecules are not only ineffective inside biological fluids but they may also accumulate inside an organism. Increasing the solubility by increasing the hydro­philicity of potentially bioactive mol­ecules may be achieved by converting them into salts (Smith *et al.*, 2009 ▸). Therefore, the synthesis of readily soluble ammonium salts of dicoumarol derivatives is of considerable importance. Herein, a crystallographically characterized example (being only the fourth of its kind) is discussed with a focus on its structural aspects.
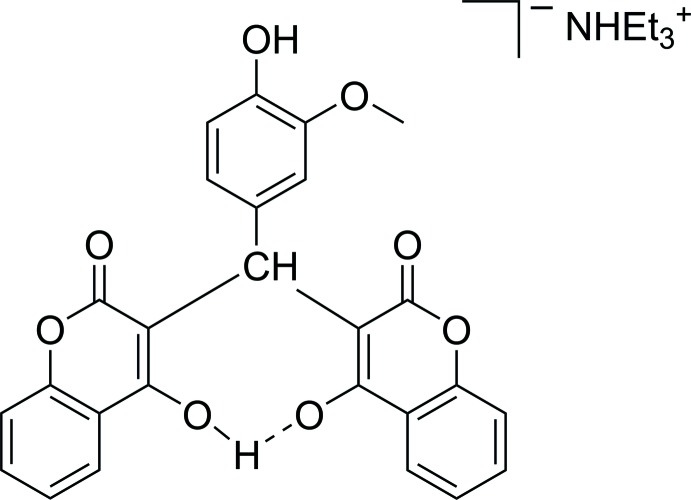


## Structural commentary   

**Figure 1:**
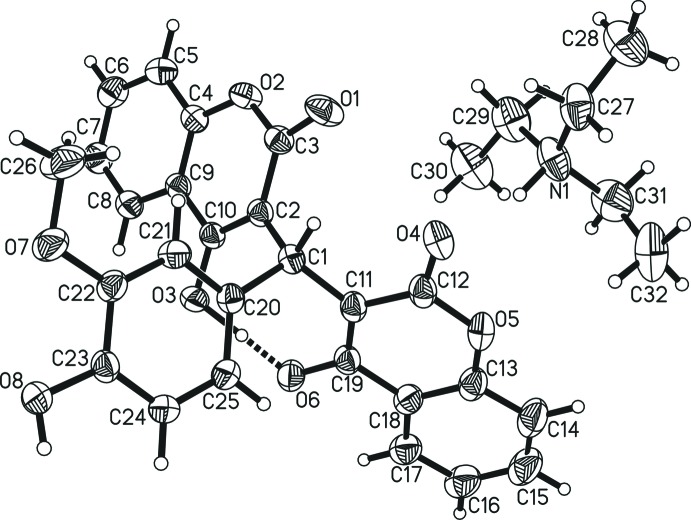
The mol­ecular structure of tri­ethyl­ammonium 3-[(4-hy­droxy-3-meth­oxy­phen­yl)(4-hy­droxy-2-oxo-2*H*-chro­men-3-yl)meth­yl]-2-oxo-2*H*-chromen-4-olate. Displacement ellipsoids are shown at the 50% probability level.

The mol­ecular structure of the title compound is shown in Fig. 1 ▸. The deprotonation of one hy­droxy-coumarin substit­uent but not the other leads to a short intra­molecular negative charge-assisted hydrogen bond between the two hy­droxy-coumarin substituents. The formation of such intra­molecular hydrogen bonds between hy­droxy-coumarin substituents is rare though not unprecedented (Kolos *et al.*, 2007 ▸; Vijayalakshmi *et al.*, 2001 ▸; Waheed & Ahmed, 2016 ▸). Recently, Bengiat and coworkers surveyed the occurrence of negative charge-assisted hydrogen bonds (–CAHB) in the Cambridge Structural Database (Groom *et al.*, 2016 ▸) in general (Bengiat *et al.*, 2016*a* ▸), covering 19 such compounds although excluding the report by Waheed & Ahmed (2016 ▸), which was published later that year. Bengiat *et al.* (2016*b* ▸) also discovered the shortest distance between donor and acceptor oxygen atoms of such inter­molecular inter­actions to be 2.404 (3) Å, whereas in all other examples the distance was given as at least 2.430 Å (Bengiat *et al.*, 2016*a* ▸). The metrical parameters of the intra­molecular –CAHB in the title compound are *D⋯A* 2.4139 (15) Å and *D*—H⋯*A* 169 (2)°. The distance of the freely refined hydrogen atom to its parent atom O3 is elong­ated to 1.18 (3) Å, while the H⋯*A* hydrogen-bond length to O6 is rather short at only 1.24 (3) Å. This inter­action is therefore the second shortest such –CAHB overall and the shortest intra­molecular one. In the three related deprotonated dicoumarols, the *D*⋯*A* distances range from 2.423 Å (Waheed & Ahmed, 2016 ▸) to 2.491 Å (Kolos *et al.*, 2007 ▸). Based on the short, and hence strong, intra­molecular hydrogen bond, an eight membered ring is formed (C1/C2/C10/O3/H3*O*/O6/C19/C11). The distances between the alcohol oxygen atoms and bound carbon atoms are 1.3005 (16) Å (O3—C10) and 1.2939 (17) Å (O6—C19); *i.e.* both very similar and both significantly shorter than those reported for non-deprotonated derivatives, which range from 1.331 to 1.338 Å (Stanchev *et al.*, 2007 ▸). This is in accordance with both alcohol functions being deprotonated and proton­ated to a certain extent at the same time, as was also found in one related structure of a salt (Vijayalakshmi *et al.*, 2001 ▸) but not in the other two analogous structures (Kolos *et al.*, 2007 ▸; Waheed & Ahmed, 2016 ▸).

The ammonium hydrogen atom, which was refined freely, exhibits a hydrogen bond to the carbonyl oxygen atom of the deprotonated coumarol substituent (N1—H1*N*⋯O4) with *D⋯A* = 2.7727 (19) Å and *D-*–H⋯*A* = 164.5 (18)°.

All of the C—C1—C angles around the central methine carbon atom [C11—C1—C2 = 116.48 (12), C11—C1—C20 = 114.44 (12), C2—C1—C20 = 110.79 (11)°] are slightly widened compared to the ideal tetra­hedral value. As this is most pronounced for the angle involving the two coumarin substit­uents, it is most likely based on steric strain. The bond lengths involving the two pyran oxygen atoms [O2—C3 = 1.3773 (18), O2—C4 =1.3692 (17), O5—C12 = 1.3789 (18) and O5—C13 = 1.365 (2) Å] are similar as observed previously, indicating conjugation between the six-membered rings in the two benzo­pyran systems (Alcock & Hough, 1972 ▸; Vijayalakshmi *et al.*, 2001 ▸). The planarity of the two benzo­pyran moieties (C2/C3/O2/C4–C10, and C11/C12/O5/C13–C19) support this conclusion, with the largest deviations from the planes found for C2 [0.089 (1) Å; carbon atom binding the central methine carbon C1] and for C18 [0.020 (1) Å]. The dihedral angle between these planes is 50.84 (4)° and they form angles with the phenyl ring plane of 76.24 (5) and 59.40 (5)°, respectively.

Notable differences to the neutral parent mol­ecule (Bandyopadhyay, 2015 ▸) comprise (i) the orientation of the hy­droxy coumarin substituents (in the neutral structure one is flipped so that the lactone and alcohol moieties face each other, whereas in the present case alcohol faces alcohol and lactone faces lactone), (ii) a contraction [1.516 (2) Å, C1—C11] and elongation [1.5277 (19) Å, C1—C2] of the methine-to-benzo­pyran-carbon-atom distances of the deprotonated and non-deprotonated substituents compared to the neutral structure (1.520 and 1.521 Å) and (iii) a higher mol­ecular symmetry including the orientation of the 4-hy­droxy-3-meth­oxy­phenyl substituent of the neutral mol­ecule compared to the anion of the title compound, emphasized by the torsion angles between the phenyl moiety and the two benzo­pyrane moieties, which are much more distinct in the anion [C2—C1—C20—C25 = 124.22 (15) and C11—C1—C20—C21 = 169.11 (13)° *vs* 153.28 and 163.81° in the neutral mol­ecule].

## Supra­molecular features   

The crystal packing appears to be dominated by inter­molecular hydrogen-bonding inter­actions. No parallel alignments of the aromatic systems (phenyl, benzo­pyran) in a stacking fashion are observed, *i.e*. π–π inter­actions are not present.

**Figure 2:**
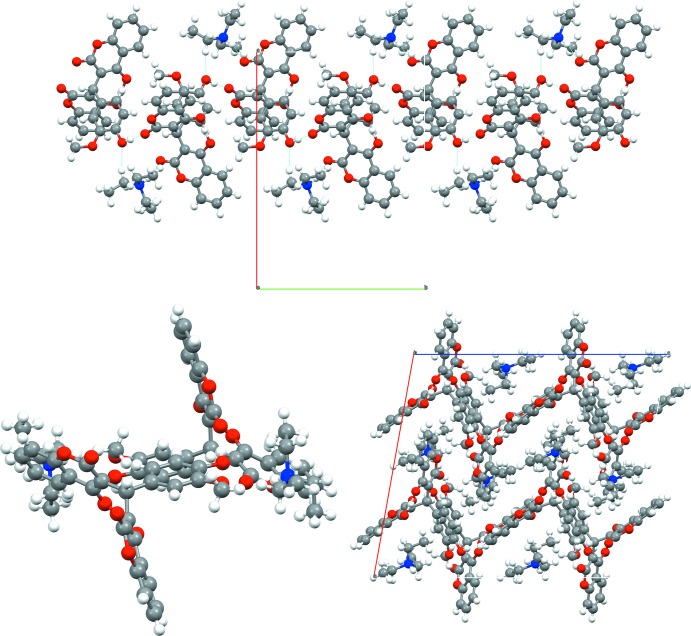
Hydrogen-bonding inter­actions forming infinite flat chains protruding along *b* viewed along *c* (top) and along *b* (bottom, left; showing the benzo­pyran moieties sticking out up and down). The crystal packing exhibiting a zigzag pattern viewed along *b* (bottom, right).

The alcohol oxygen atom of the 4-hy­droxy-3-meth­oxy­phenyl substituent (O8) bridges the adjacent cation and anion by hydrogen bonding as a classical donor [O8—H8*O*⋯O1(−*x* + 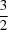, *y* + 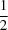, −*z* + 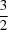); *D*⋯*A* = 2.4139 (15) Å] and as acceptor [O8⋯H27*B*—C27(−*x* + 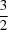, *y* + 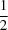, −*z* + 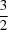); *D*⋯*A* = 3.257 (2) Å] in a non-classical hydrogen bond from an amine methyl group (Table 1 ▸; Fig. 2 ▸, top). The ammonium cations bridge adjacent anions by the intra-formula classical hydrogen bond (N1—H1*N*⋯O4; see above) and the non-classical donation towards O8 [C27—H27*B*⋯O8(−*x* + 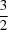, *y* − 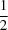, −*z* + 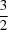; *D⋯A =* 3.257 (2) (19) Å]. Supported by the hydrogen bond with the carbonyl oxygen atom O1 as acceptor [O1⋯H8*O*—O8(−*x* + 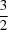, *y* − 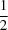, −*z* + 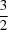); *D*⋯*A* = 2.6488 (16) Å], these inter­actions form infinite flat chains with ‘up and down’-pointing benzo­pyrane moieties protruding along **b** (Fig. 2 ▸, bottom left). The packing diagram exhibits a zigzag pattern along **b** in which adjacent chains are aligned in a zipper-like fashion (Fig. 2 ▸, bottom right).

## Synthesis and crystallization   

3,3′-[(3-Meth­oxy-4-hy­droxy­phen­yl)methanedi­yl]bis­(4-hy­droxy-2*H*-chromen-2-one) was synthesized following essentially the reported procedure (Rehman *et al.*, 2013 ▸). 20 mmol of 3-meth­oxy-4-hy­droxy­benzaldehyde dissolved in anyhydrous ethanol was added to 50 mmol of an ethano­lic solution of 4-hy­droxy­coumarin. The resulting mixture was refluxed at 393 K for 3 h. Upon cooling, a solid white powder was obtained, which was washed with 10% copious ethano­lic/*n*-hexane solution. The subsequent deprotonation of 3,3′-[(3-meth­oxy-4-hy­droxy­phen­yl)methanedi­yl]bis­(4-hy­droxy-2*H*-chromen-2-one) was carried out by adding 1 mL of tri­ethyl­amine to its methano­lic solution. The resulting transparent yellowish solution was left standing overnight to grow transparent crystals of tri­ethyl­ammonium 3-[(4-hy­droxy-3-meth­oxy­phen­yl)(4-hy­droxy-2-oxo-2*H*-chromen-3-yl)meth­yl]-2-oxo-2*H*-chromen-4-olate.

## Refinement   

Crystal data, data collection and structure refinement details are summarized in Table 2 ▸. The three hydrogen atoms bound to heteroatoms (N1, O3, O8) were freely refined. Carbon-bound hydrogen atoms were placed in calculated positions, and refined with a riding-model approximation: C—H = 0.95–1.00 Å with *U*_iso_(H) = 1.5*U*_eq_(C-methyl) and 1.2*U*_eq_(C) for other H atoms.

Hydrogen-bonding inter­actions were identified and analysed using *PLATON* (Spek, 2009 ▸) and finally calculated using the HTAB instruction in *SHELXL* (together with EQIV) (Sheldrick, 2015*b* ▸).

## Supplementary Material

Crystal structure: contains datablock(s) I. DOI: 10.1107/S2056989018001561/lh5866sup1.cif

Structure factors: contains datablock(s) I. DOI: 10.1107/S2056989018001561/lh5866Isup2.hkl

Supporting information file. DOI: 10.1107/S2056989018001561/lh5866Isup3.cml

CCDC reference: 1818945

Additional supporting information:  crystallographic information; 3D view; checkCIF report

## Figures and Tables

**Table 1:** Hydrogen-bond geometry (Å, °)

*D*—H⋯*A*	*D*—H	H⋯*A*	*D*⋯*A*	*D*—H⋯*A*
O3—H3*O*⋯O6	1.18 (3)	1.24 (3)	2.4139 (15)	169 (2)
O8—H8*O*⋯O1^i^	0.869 (19)	1.789 (19)	2.6488 (16)	170.0 (18)
C27—H27*B*⋯O8^ii^	0.99	2.31	3.257 (2)	161
N1—H1*N*⋯O4	0.98 (2)	1.82 (2)	2.7727 (19)	164.5 (18)

Symmetry codes: (i) 
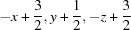
; (ii) 
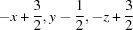
.

**Table 2:** Experimental details

Crystal data	
Chemical formula	C_6_H_16_N^+^·C_26_H_17_O_8_^−^
*M*_r_	559.59
Crystal system, space group	Monoclinic, *C*2/*c*
Temperature (K)	170
*a*, *b*, *c* (Å)	19.408 (4), 13.518 (3), 21.714 (4)
β (°)	100.16 (3)
*V* (Å^3^)	5607 (2)
*Z*	8
Radiation type	Mo *K*α
μ (mm^−1^)	0.10
Crystal size (mm)	0.44 × 0.39 × 0.37

Data collection	
Diffractometer	Stoe IPDS2T
Absorption correction	Numerical (*X-RED32* and *X-SHAPE*; Stoe & Cie, 2010 ▸)
*T*_min_, *T*_max_	0.784, 0.927
No. of measured, independent and observed [*I* > 2σ(*I*)] reflections	31033, 7726, 4433
*R*_int_	0.054
(sin θ/λ)_max_ (Å^−1^)	0.695

Refinement	
*R*[*F* ^2^ > 2σ(*F* ^2^)], *wR*(*F* ^2^), *S*	0.044, 0.122, 0.90
No. of reflections	7726
No. of parameters	386
H-atom treatment	H atoms treated by a mixture of independent and constrained refinement
Δρ_max_, Δρ_min_ (e Å^−3^)	0.44, −0.27

Computer programs: *X-AREA* (Stoe & Cie, 2010 ▸), *SHELXT2016* (Sheldrick, 2015*a* ▸), *SHELXL2016* (Sheldrick, 2015*b* ▸), *XP* in *SHELXTL* (Sheldrick, 2008 ▸), *CIFTAB* (Sheldrick, 2008 ▸) and *Mercury* (Macrae *et al.*, 2006 ▸).

## supplementary crystallographic information

### Crystal data

**Table:**

C_6_H_16_N^+^·C_26_H_17_O_8_^−^	*F*(000) = 2368
*M**_r_* = 559.59	*D*_x_ = 1.326 Mg m^−^^3^
Monoclinic, *C*2/*c*	Mo *K*α radiation, λ = 0.71073 Å
*a* = 19.408 (4) Å	Cell parameters from 31667 reflections
*b* = 13.518 (3) Å	θ = 6.3–59.2°
*c* = 21.714 (4) Å	µ = 0.10 mm^−^^1^
β = 100.16 (3)°	*T* = 170 K
*V* = 5607 (2) Å^3^	Prism, colourless
*Z* = 8	0.44 × 0.39 × 0.37 mm

### Data collection

**Table:**

Stoe IPDS2T diffractometer	7726 independent reflections
Radiation source: fine-focus sealed tube	4433 reflections with *I* > 2σ(*I*)
Detector resolution: 6.67 pixels mm^-1^	*R*_int_ = 0.054
ω scans	θ_max_ = 29.6°, θ_min_ = 3.2°
Absorption correction: numerical (X-RED32 and X-SHAPE; Stoe & Cie, 2010)	*h* = −26→26
*T*_min_ = 0.784, *T*_max_ = 0.927	*k* = −18→18
31033 measured reflections	*l* = −29→30

### Refinement

**Table:**

Refinement on *F*^2^	0 restraints
Least-squares matrix: full	Hydrogen site location: mixed
*R*[*F*^2^ > 2σ(*F*^2^)] = 0.044	H atoms treated by a mixture of independent and constrained refinement
*wR*(*F*^2^) = 0.122	*w* = 1/[σ^2^(*F*_o_^2^) + (0.0718*P*)^2^] where *P* = (*F*_o_^2^ + 2*F*_c_^2^)/3
*S* = 0.90	(Δ/σ)_max_ < 0.001
7726 reflections	Δρ_max_ = 0.44 e Å^−^^3^
386 parameters	Δρ_min_ = −0.26 e Å^−^^3^

### Special details

**Table:**

Geometry. All esds (except the esd in the dihedral angle between two l.s. planes) are estimated using the full covariance matrix. The cell esds are taken into account individually in the estimation of esds in distances, angles and torsion angles; correlations between esds in cell parameters are only used when they are defined by crystal symmetry. An approximate (isotropic) treatment of cell esds is used for estimating esds involving l.s. planes.

### Fractional atomic coordinates and isotropic or equivalent isotropic displacement parameters (Å^2^)

**Table:**

	*x*	*y*	*z*	*U*_iso_*/*U*_eq_	
O1	0.65404 (7)	0.33340 (8)	0.59806 (5)	0.0450 (3)	
O2	0.71609 (6)	0.37291 (7)	0.52747 (5)	0.0351 (2)	
O3	0.72176 (6)	0.66489 (7)	0.57698 (5)	0.0349 (2)	
O4	0.54117 (6)	0.42562 (8)	0.68855 (5)	0.0392 (3)	
O5	0.46745 (5)	0.54780 (8)	0.66496 (5)	0.0413 (3)	
O6	0.61390 (6)	0.72322 (8)	0.60507 (5)	0.0380 (3)	
O7	0.89787 (6)	0.52052 (8)	0.78148 (5)	0.0432 (3)	
O8	0.88267 (5)	0.69683 (8)	0.82643 (5)	0.0382 (3)	
C1	0.65911 (7)	0.52333 (10)	0.65646 (6)	0.0283 (3)	
H1	0.653226	0.456518	0.674616	0.034*	
C2	0.68601 (7)	0.50216 (10)	0.59576 (6)	0.0274 (3)	
C3	0.68308 (8)	0.40112 (10)	0.57585 (6)	0.0307 (3)	
C4	0.75438 (8)	0.43890 (10)	0.49954 (7)	0.0304 (3)	
C5	0.79254 (9)	0.40104 (12)	0.45636 (7)	0.0380 (3)	
H5	0.791402	0.332382	0.446889	0.046*	
C6	0.83190 (9)	0.46504 (12)	0.42771 (7)	0.0397 (4)	
H6	0.859256	0.440327	0.398885	0.048*	
C7	0.83193 (9)	0.56603 (12)	0.44068 (7)	0.0391 (4)	
H7	0.858278	0.609993	0.419785	0.047*	
C8	0.79410 (8)	0.60231 (11)	0.48347 (7)	0.0340 (3)	
H8	0.794209	0.671288	0.491881	0.041*	
C9	0.75550 (7)	0.53842 (10)	0.51466 (6)	0.0282 (3)	
C10	0.71880 (7)	0.57089 (10)	0.56401 (6)	0.0280 (3)	
C11	0.58739 (7)	0.57065 (11)	0.64990 (6)	0.0306 (3)	
C12	0.53443 (8)	0.51111 (11)	0.66936 (7)	0.0334 (3)	
C13	0.45011 (8)	0.64011 (12)	0.64179 (7)	0.0384 (4)	
C14	0.38105 (9)	0.66885 (15)	0.63775 (9)	0.0515 (4)	
H14	0.347869	0.626144	0.651362	0.062*	
C15	0.36156 (10)	0.76085 (16)	0.61355 (10)	0.0577 (5)	
H15	0.314319	0.781672	0.610423	0.069*	
C16	0.40959 (9)	0.82347 (15)	0.59370 (9)	0.0517 (5)	
H16	0.395311	0.886758	0.577164	0.062*	
C17	0.47810 (9)	0.79387 (12)	0.59795 (8)	0.0420 (4)	
H17	0.511084	0.836923	0.584344	0.050*	
C18	0.49943 (8)	0.70091 (11)	0.62216 (7)	0.0352 (3)	
C19	0.57071 (7)	0.66420 (11)	0.62576 (7)	0.0313 (3)	
C20	0.71563 (7)	0.57348 (10)	0.70423 (6)	0.0282 (3)	
C21	0.77965 (8)	0.52424 (10)	0.72074 (7)	0.0315 (3)	
H21	0.785784	0.461518	0.702725	0.038*	
C22	0.83384 (7)	0.56465 (11)	0.76247 (6)	0.0313 (3)	
C23	0.82595 (7)	0.65759 (11)	0.78824 (6)	0.0303 (3)	
C24	0.76243 (8)	0.70469 (11)	0.77388 (7)	0.0338 (3)	
H24	0.756016	0.766884	0.792446	0.041*	
C25	0.70744 (8)	0.66260 (11)	0.73256 (7)	0.0331 (3)	
H25	0.663662	0.695791	0.723776	0.040*	
C26	0.90262 (10)	0.41779 (13)	0.76835 (9)	0.0543 (5)	
H26A	0.865134	0.382110	0.783783	0.081*	
H26B	0.948123	0.392467	0.789170	0.081*	
H26C	0.897890	0.408094	0.723074	0.081*	
H3O	0.6704 (13)	0.6889 (17)	0.5951 (11)	0.085 (7)*	
H8O	0.8688 (10)	0.7456 (14)	0.8472 (9)	0.047 (5)*	
N1	0.43844 (8)	0.30039 (12)	0.62696 (7)	0.0470 (4)	
C27	0.44835 (9)	0.20824 (14)	0.66564 (9)	0.0509 (5)	
H27A	0.431136	0.220287	0.705272	0.061*	
H27B	0.499039	0.193640	0.676315	0.061*	
C28	0.41125 (13)	0.11883 (15)	0.63391 (11)	0.0694 (6)	
H28A	0.360606	0.130354	0.626276	0.104*	
H28B	0.422180	0.060744	0.660876	0.104*	
H28C	0.426885	0.107345	0.593994	0.104*	
C29	0.46749 (11)	0.28595 (16)	0.56641 (9)	0.0583 (5)	
H29A	0.512240	0.249257	0.576127	0.070*	
H29B	0.434245	0.244931	0.537270	0.070*	
C30	0.47967 (13)	0.38019 (16)	0.53470 (9)	0.0651 (6)	
H30A	0.434859	0.413819	0.520966	0.098*	
H30B	0.501585	0.366045	0.498311	0.098*	
H30C	0.510589	0.422827	0.563915	0.098*	
C31	0.36666 (10)	0.34013 (16)	0.61423 (10)	0.0604 (5)	
H31A	0.334931	0.289439	0.591806	0.072*	
H31B	0.365407	0.398452	0.586472	0.072*	
C32	0.34037 (11)	0.3698 (2)	0.67264 (10)	0.0723 (7)	
H32A	0.331448	0.310448	0.695904	0.108*	
H32B	0.296877	0.407609	0.661328	0.108*	
H32C	0.375635	0.410813	0.698784	0.108*	
H1N	0.4677 (11)	0.3515 (15)	0.6501 (10)	0.064 (6)*	

### Atomic displacement parameters (Å^2^)

**Table:**

	*U*^11^	*U*^22^	*U*^33^	*U*^12^	*U*^13^	*U*^23^
O1	0.0646 (8)	0.0275 (5)	0.0459 (6)	−0.0090 (5)	0.0180 (6)	0.0021 (5)
O2	0.0467 (6)	0.0250 (5)	0.0350 (5)	−0.0026 (4)	0.0105 (5)	−0.0019 (4)
O3	0.0372 (6)	0.0243 (5)	0.0460 (6)	−0.0043 (4)	0.0146 (5)	−0.0036 (4)
O4	0.0366 (6)	0.0435 (6)	0.0367 (5)	−0.0117 (5)	0.0039 (4)	0.0044 (5)
O5	0.0263 (5)	0.0525 (7)	0.0458 (6)	−0.0047 (5)	0.0080 (5)	0.0017 (5)
O6	0.0306 (6)	0.0333 (5)	0.0511 (6)	0.0003 (4)	0.0094 (5)	0.0053 (5)
O7	0.0334 (6)	0.0446 (6)	0.0470 (6)	0.0127 (5)	−0.0056 (5)	−0.0110 (5)
O8	0.0284 (6)	0.0417 (6)	0.0427 (6)	−0.0011 (5)	0.0011 (5)	−0.0143 (5)
C1	0.0270 (7)	0.0281 (7)	0.0295 (7)	−0.0029 (5)	0.0042 (5)	0.0015 (5)
C2	0.0250 (7)	0.0277 (6)	0.0282 (6)	−0.0012 (5)	0.0012 (5)	0.0000 (5)
C3	0.0330 (8)	0.0278 (7)	0.0300 (7)	−0.0009 (6)	0.0023 (6)	0.0018 (5)
C4	0.0316 (7)	0.0284 (7)	0.0299 (7)	−0.0019 (6)	0.0021 (6)	0.0012 (5)
C5	0.0440 (9)	0.0333 (8)	0.0368 (8)	0.0032 (7)	0.0074 (7)	−0.0039 (6)
C6	0.0410 (9)	0.0470 (9)	0.0326 (8)	0.0028 (7)	0.0104 (7)	−0.0022 (6)
C7	0.0401 (9)	0.0413 (9)	0.0373 (8)	−0.0039 (7)	0.0105 (7)	0.0012 (6)
C8	0.0344 (8)	0.0315 (7)	0.0362 (7)	−0.0028 (6)	0.0068 (6)	0.0004 (6)
C9	0.0266 (7)	0.0275 (6)	0.0289 (7)	−0.0003 (5)	0.0002 (5)	0.0008 (5)
C10	0.0255 (7)	0.0258 (6)	0.0311 (7)	−0.0009 (5)	0.0008 (5)	−0.0003 (5)
C11	0.0256 (7)	0.0362 (7)	0.0292 (7)	−0.0039 (6)	0.0031 (5)	−0.0028 (6)
C12	0.0283 (7)	0.0427 (8)	0.0284 (7)	−0.0058 (6)	0.0031 (6)	−0.0041 (6)
C13	0.0278 (8)	0.0513 (9)	0.0352 (8)	−0.0002 (7)	0.0032 (6)	−0.0065 (7)
C14	0.0292 (8)	0.0706 (12)	0.0546 (10)	0.0016 (8)	0.0072 (7)	−0.0067 (9)
C15	0.0303 (9)	0.0755 (13)	0.0647 (12)	0.0120 (9)	0.0010 (8)	−0.0129 (10)
C16	0.0396 (10)	0.0584 (11)	0.0526 (10)	0.0135 (9)	−0.0045 (8)	−0.0088 (8)
C17	0.0366 (9)	0.0457 (9)	0.0414 (8)	0.0059 (7)	0.0003 (7)	−0.0054 (7)
C18	0.0285 (8)	0.0433 (8)	0.0318 (7)	0.0017 (6)	−0.0001 (6)	−0.0067 (6)
C19	0.0271 (7)	0.0347 (7)	0.0313 (7)	−0.0037 (6)	0.0032 (6)	−0.0034 (6)
C20	0.0268 (7)	0.0301 (7)	0.0280 (6)	−0.0018 (6)	0.0055 (5)	0.0003 (5)
C21	0.0345 (8)	0.0289 (7)	0.0302 (7)	0.0038 (6)	0.0028 (6)	−0.0027 (5)
C22	0.0266 (7)	0.0355 (7)	0.0307 (7)	0.0043 (6)	0.0023 (5)	−0.0012 (6)
C23	0.0260 (7)	0.0351 (7)	0.0288 (7)	−0.0037 (6)	0.0025 (5)	−0.0033 (6)
C24	0.0315 (8)	0.0311 (7)	0.0385 (8)	0.0007 (6)	0.0056 (6)	−0.0081 (6)
C25	0.0261 (7)	0.0340 (7)	0.0384 (8)	0.0030 (6)	0.0037 (6)	−0.0033 (6)
C26	0.0536 (11)	0.0461 (10)	0.0575 (11)	0.0231 (9)	−0.0055 (9)	−0.0063 (8)
N1	0.0418 (8)	0.0546 (8)	0.0391 (7)	−0.0160 (7)	−0.0081 (6)	0.0083 (6)
C27	0.0362 (9)	0.0638 (12)	0.0495 (10)	−0.0042 (8)	−0.0010 (8)	0.0164 (9)
C28	0.0777 (15)	0.0541 (12)	0.0745 (14)	−0.0127 (11)	0.0082 (12)	0.0119 (10)
C29	0.0603 (12)	0.0710 (13)	0.0402 (9)	−0.0151 (10)	−0.0002 (8)	−0.0013 (9)
C30	0.0820 (15)	0.0759 (14)	0.0356 (9)	−0.0231 (12)	0.0051 (9)	0.0010 (9)
C31	0.0511 (11)	0.0590 (12)	0.0622 (12)	−0.0008 (9)	−0.0145 (9)	0.0007 (10)
C32	0.0476 (12)	0.1114 (19)	0.0579 (12)	−0.0237 (12)	0.0092 (10)	0.0128 (12)

### Geometric parameters (Å, º)

**Table:**

O1—C3	1.2183 (17)	C16—C17	1.376 (2)
O2—C4	1.3692 (17)	C16—H16	0.9500
O2—C3	1.3773 (18)	C17—C18	1.396 (2)
O3—C10	1.3005 (16)	C17—H17	0.9500
O3—H3O	1.18 (3)	C18—C19	1.459 (2)
O4—C12	1.2275 (18)	C20—C25	1.375 (2)
O5—C13	1.365 (2)	C20—C21	1.399 (2)
O5—C12	1.3789 (18)	C21—C22	1.375 (2)
O6—C19	1.2939 (17)	C21—H21	0.9500
O6—H3O	1.24 (3)	C22—C23	1.395 (2)
O7—C22	1.3745 (17)	C23—C24	1.374 (2)
O7—C26	1.424 (2)	C24—C25	1.389 (2)
O8—C23	1.3634 (17)	C24—H24	0.9500
O8—H8O	0.869 (19)	C25—H25	0.9500
C1—C11	1.516 (2)	C26—H26A	0.9800
C1—C2	1.5277 (19)	C26—H26B	0.9800
C1—C20	1.5287 (19)	C26—H26C	0.9800
C1—H1	1.0000	N1—C31	1.473 (2)
C2—C10	1.3779 (19)	N1—C27	1.496 (2)
C2—C3	1.4307 (19)	N1—C29	1.532 (3)
C4—C9	1.384 (2)	N1—H1N	0.98 (2)
C4—C5	1.391 (2)	C27—C28	1.510 (3)
C5—C6	1.374 (2)	C27—H27A	0.9900
C5—H5	0.9500	C27—H27B	0.9900
C6—C7	1.394 (2)	C28—H28A	0.9800
C6—H6	0.9500	C28—H28B	0.9800
C7—C8	1.372 (2)	C28—H28C	0.9800
C7—H7	0.9500	C29—C30	1.487 (3)
C8—C9	1.395 (2)	C29—H29A	0.9900
C8—H8	0.9500	C29—H29B	0.9900
C9—C10	1.455 (2)	C30—H30A	0.9800
C11—C19	1.385 (2)	C30—H30B	0.9800
C11—C12	1.427 (2)	C30—H30C	0.9800
C13—C14	1.383 (2)	C31—C32	1.503 (3)
C13—C18	1.385 (2)	C31—H31A	0.9900
C14—C15	1.377 (3)	C31—H31B	0.9900
C14—H14	0.9500	C32—H32A	0.9800
C15—C16	1.383 (3)	C32—H32B	0.9800
C15—H15	0.9500	C32—H32C	0.9800
			
C4—O2—C3	121.30 (11)	C25—C20—C21	118.02 (13)
C10—O3—H3O	109.5 (11)	C25—C20—C1	124.51 (13)
C13—O5—C12	121.49 (12)	C21—C20—C1	117.47 (12)
C19—O6—H3O	118.4 (11)	C22—C21—C20	121.43 (13)
C22—O7—C26	116.76 (13)	C22—C21—H21	119.3
C23—O8—H8O	108.6 (12)	C20—C21—H21	119.3
C11—C1—C2	116.48 (12)	O7—C22—C21	124.85 (13)
C11—C1—C20	114.44 (12)	O7—C22—C23	115.34 (12)
C2—C1—C20	110.79 (11)	C21—C22—C23	119.80 (13)
C11—C1—H1	104.5	O8—C23—C24	123.63 (13)
C2—C1—H1	104.5	O8—C23—C22	117.41 (13)
C20—C1—H1	104.5	C24—C23—C22	118.96 (13)
C10—C2—C3	119.32 (13)	C23—C24—C25	120.88 (13)
C10—C2—C1	124.31 (12)	C23—C24—H24	119.6
C3—C2—C1	115.99 (12)	C25—C24—H24	119.6
O1—C3—O2	113.83 (12)	C20—C25—C24	120.78 (13)
O1—C3—C2	126.45 (14)	C20—C25—H25	119.6
O2—C3—C2	119.72 (12)	C24—C25—H25	119.6
O2—C4—C9	121.00 (13)	O7—C26—H26A	109.5
O2—C4—C5	117.01 (12)	O7—C26—H26B	109.5
C9—C4—C5	121.98 (14)	H26A—C26—H26B	109.5
C6—C5—C4	118.58 (14)	O7—C26—H26C	109.5
C6—C5—H5	120.7	H26A—C26—H26C	109.5
C4—C5—H5	120.7	H26B—C26—H26C	109.5
C5—C6—C7	120.38 (15)	C31—N1—C27	115.64 (15)
C5—C6—H6	119.8	C31—N1—C29	111.38 (15)
C7—C6—H6	119.8	C27—N1—C29	110.21 (15)
C8—C7—C6	120.37 (15)	C31—N1—H1N	106.5 (12)
C8—C7—H7	119.8	C27—N1—H1N	107.2 (12)
C6—C7—H7	119.8	C29—N1—H1N	105.2 (12)
C7—C8—C9	120.32 (14)	N1—C27—C28	114.01 (15)
C7—C8—H8	119.8	N1—C27—H27A	108.8
C9—C8—H8	119.8	C28—C27—H27A	108.8
C4—C9—C8	118.30 (14)	N1—C27—H27B	108.8
C4—C9—C10	118.59 (13)	C28—C27—H27B	108.8
C8—C9—C10	123.06 (13)	H27A—C27—H27B	107.6
O3—C10—C2	123.86 (13)	C27—C28—H28A	109.5
O3—C10—C9	116.45 (12)	C27—C28—H28B	109.5
C2—C10—C9	119.63 (12)	H28A—C28—H28B	109.5
C19—C11—C12	119.64 (13)	C27—C28—H28C	109.5
C19—C11—C1	124.77 (13)	H28A—C28—H28C	109.5
C12—C11—C1	115.57 (13)	H28B—C28—H28C	109.5
O4—C12—O5	113.87 (13)	C30—C29—N1	113.64 (17)
O4—C12—C11	126.33 (14)	C30—C29—H29A	108.8
O5—C12—C11	119.74 (13)	N1—C29—H29A	108.8
O5—C13—C14	116.94 (15)	C30—C29—H29B	108.8
O5—C13—C18	121.14 (14)	N1—C29—H29B	108.8
C14—C13—C18	121.90 (16)	H29A—C29—H29B	107.7
C15—C14—C13	118.40 (18)	C29—C30—H30A	109.5
C15—C14—H14	120.8	C29—C30—H30B	109.5
C13—C14—H14	120.8	H30A—C30—H30B	109.5
C14—C15—C16	121.12 (17)	C29—C30—H30C	109.5
C14—C15—H15	119.4	H30A—C30—H30C	109.5
C16—C15—H15	119.4	H30B—C30—H30C	109.5
C17—C16—C15	119.84 (18)	N1—C31—C32	112.94 (17)
C17—C16—H16	120.1	N1—C31—H31A	109.0
C15—C16—H16	120.1	C32—C31—H31A	109.0
C16—C17—C18	120.38 (18)	N1—C31—H31B	109.0
C16—C17—H17	119.8	C32—C31—H31B	109.0
C18—C17—H17	119.8	H31A—C31—H31B	107.8
C13—C18—C17	118.36 (15)	C31—C32—H32A	109.5
C13—C18—C19	118.82 (14)	C31—C32—H32B	109.5
C17—C18—C19	122.80 (15)	H32A—C32—H32B	109.5
O6—C19—C11	124.88 (13)	C31—C32—H32C	109.5
O6—C19—C18	115.97 (13)	H32A—C32—H32C	109.5
C11—C19—C18	119.14 (13)	H32B—C32—H32C	109.5

### Hydrogen-bond geometry (Å, º)

**Table:**

*D*—H···*A*	*D*—H	H···*A*	*D*···*A*	*D*—H···*A*
O3—H3*O*···O6	1.18 (3)	1.24 (3)	2.4139 (15)	169 (2)
O8—H8*O*···O1^i^	0.869 (19)	1.789 (19)	2.6488 (16)	170.0 (18)
C27—H27*B*···O8^ii^	0.99	2.31	3.257 (2)	161
N1—H1*N*···O4	0.98 (2)	1.82 (2)	2.7727 (19)	164.5 (18)

Symmetry codes: (i) −*x*+3/2, *y*+1/2, −*z*+3/2; (ii) −*x*+3/2, *y*−1/2, −*z*+3/2.
